# Acute Gastroenteritis Associated with Norovirus GII.8[P8], Thailand, 2023

**DOI:** 10.3201/eid3001.231264

**Published:** 2024-01

**Authors:** Watchaporn Chuchaona, Sompong Vongpunsawad, Weerasak Lawtongkum, Nattawan Thepnarong, Yong Poovorawan

**Affiliations:** Chulalongkorn University, Bangkok, Thailand (W. Chuchaona, S. Vongpunsawad, Y. Poovorawan);; Vachira Phuket Hospital, Phuket, Thailand (W. Lawtongkum, N. Thepnarong)

**Keywords:** norovirus, gastroenteritis, viruses, food safety, GII.8, Thailand, VP1, polymerase, enteric infections

## Abstract

Acute gastroenteritis associated with human norovirus infection was reported in Phuket, Thailand, in June 2023. We amplified GII.8[P8] from the outbreak stool specimens. Retrospective sample analysis identified infrequent GII.8[P8] in the country beginning in 2018. In all, the 10 whole-genome GII.8[P8] sequences from Thailand we examined had no evidence of genotypic recombination.

Norovirus is the most common cause of acute viral gastroenteritis among adults and children and has no currently approved vaccine (*1*). Norovirus is genetically diverse and is classified into 10 genogroups (GI–GX) representing ≈50 genotypes, of which GI and GII predominantly infect humans (*2*). Currently, dual-typing of the RNA-dependent RNA polymerase (RdRp) gene in the open reading frame 1 region and the major capsid protein (VP1) gene in the open reading frame 2 region is required for proper genotype assignment and detection of viral recombinants (*3*).

In June 2023, health officials in Thailand were investigating diarrheal outbreaks that occurred on Phuket Island in southern Thailand, which is frequented by international travelers (https://www.bangkokpost.com/thailand/general/2592541/phukets-diarrhoea-outbreak-wanes-cause-still-unknown). Two stool specimens were eventually sent to our laboratory at the Center of Excellence in Clinical Virology at Chulalongkorn University (Bangkok) for molecular typing. The study was approved by Chulalongkorn University Institutional Review Board (approval no. 549/62). After viral RNA extraction from the stool specimens, quantitative real-time reverse transcription PCR (*4*) identified GII norovirus in both specimens. Confirmation assays using conventional reverse transcription PCR (*5*) with additional primers (Appendix 1 Table 1) and nucleotide sequencing yielded near-complete genomes, which we subjected to the norovirus genotyping tools of the Netherlands’ National Institute for Public Health and the Environment (https://www.rivm.nl/mpf/norovirus/typingtool) and the US Centers for Disease Control and Prevention (https://calicivirustypingtool.cdc.gov).

Both specimens from Phuket were human norovirus GII.8[P8]. Because GII.8[P8] is relatively uncommon and rarely linked to large outbreaks, we retrospectively examined archived stool specimens dating back to 2018 to determine the frequency of past infection in the country. We identified 8 additional GII.8 strains (Table), all of which were GII.8[P8]. We deposited these complete genome sequences in GenBank (accession nos. OR546391–OR546400).

**Table:** Human norovirus GII.8[P8] strains identified in Thailand, 2018–2023

Collection date	Specimen ID	Patient age, y/Sex	Location	Specimen type
2018 Feb 2	B4899	5/M	Saraburi	Stool
2018 Feb 18	B5182	7/M	Bangkok	Stool
2018 Sep 18	B6213	6/F	Nonthaburi	Stool
2019 Jul 30	B6941	12/M	Nonthaburi	Stool
2020 Feb 04	B7634	29/F	Bangkok	Stool
2023 Feb 22	B9202	3/M	Bangkok	Stool
2023 Feb 27	B9256	12/F	Chaiyaphum	Stool
2023 Apr 19	B9804	10/M	Bangkok	Stool
2023 Jun 14	B10039	12/F	Phuket	Rectal swab
2023 Jun 13	B10069	7/F	Phuket	Stool

All 10 patients who tested positive for GII.8[P8] were relatively young (age range 3–29 years, mean age 10.8 years + 7.1 SD). Five patients had vomiting and diarrhea, 3 had vomiting only, and 2 had diarrhea only (Appendix 1 Table 2). Minor symptoms were nausea, abdominal pain, fever, and headaches. All but 1 patient required 1–2 nights of hospital stay.

From the complete nucleotide sequences of the RdRp and VP1 genes, the GII.8[P8] strains from Thailand phylogenetically clustered with strains identified in Canada (GenBank accession no. MW661257), China (GenBank accession nos. MK213549 and MN996298), and the United States (GenBank accession nos. MZ292794 and OP686904) during the previous 10 years (Figure). Collectively, nucleotide sequence identities of GII.8[P8] strains from Thailand and other strains were 85%–99% over the entire genome compared with the prototypic GII.8[P8] SaitamaU25 (GenBank accession no. AB039780) (Appendix 1 Figure). However, Phuket GII.8[P8] appeared to diverge most from other GII.8[P8] strains in parts of the nonstructural protein 1–2 (p48), nonstructural protein 3 (NTPase), and VP1 shell domain.

**Figure:**
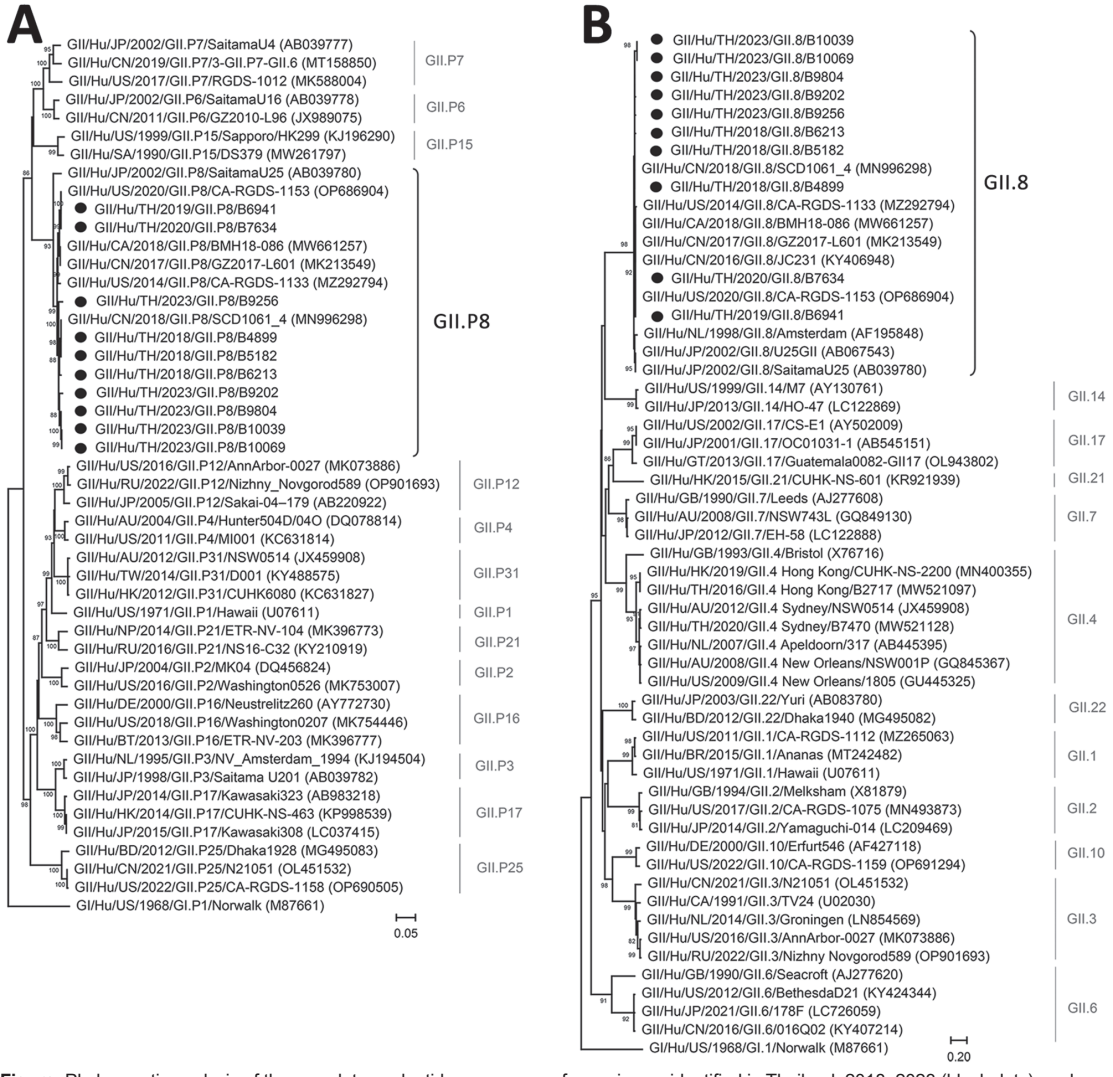
Phylogenetic analysis of the complete nucleotide sequences of noroviruses identified in Thailand, 2018–2023 (black dots), and reference sequences. A) RNA-dependent RNA polymerase (RdRp) region; B) major capsid protein (VP1) region. Trees were generated using the maximum-likelihood method based on the general time reversible model, with 1,000 bootstrap replications for branch support as implemented in MEGA software version 11 (http://www.megasoftware.net). Bootstrap values >80 are indicated at the branch nodes. GenBank accession numbers for reference sequences are provided in parentheses. Scale bar indicates nucleotide substitutions per site.

To address whether Phuket GII.8[P8] strains had developed notable amino acid changes on its genome, we compared their deduced residues to other GII.8[P8] strains. Phuket GII.8[P8] shared many unique residue changes with the most recent strain from Thailand (B9804) identified in Bangkok 2 months prior (Appendix 2 Table). No apparent mutations to suggest increased virulence or viral transmissibility were obvious, although >10 residue positions scattered throughout the GII.8[P8] genome identified in Thailand in 2023 were not shared by other known GII.8[P8] sequences. Most residue variations were conservative changes; however, T479S on VP1 is a highly conserved position among GII noroviruses.

The potential for GII.8[P8] to cause the recent norovirus outbreak in Phuket was unexpected given that the last reported outbreak in Thailand was caused by a novel GII.3[P25] recombinant in Chanthaburi Province (*6*). Of note, GII.8[P8] outbreaks are infrequent (*7*), and the most recent occurrence was foodborne (through contaminated raspberries) (*8*). No specific food source was identified and laboratory-confirmed for norovirus, and anecdotal evidence suggests probable person-to-person norovirus transmission in the Phuket outbreak. Reports of GII.8[P8] infection in the literature have not identified RpRp–VP1 recombinants, and comprehensive historical analysis of norovirus sequences suggests that GII.8 RdRp and VP1 rarely recombine with other genotypes (*9*).

Molecular analysis in this study was limited because <40 complete GII.8[P8] genomes were available in the public database. This study was also constrained by the scarcity of specimens sent for laboratory testing, which underscored limited awareness and importance placed by health officials toward timely etiologic diagnosis. A study suggests that antibodies elicited by GI.1 and GII.4 (2 genotypes in the norovirus vaccine candidate under consideration) minimally block the binding of GII.8 VLPs to histo–blood group antigens (*10*). Although unlikely, any potential increase in the prevalence of GII.8[P8] could affect real-world norovirus vaccine effectiveness. In summary, GII.8[P8] genomes identified in this study are expected to contribute to the ongoing molecular and epidemiologic surveillance of community-acquired norovirus infection, which could benefit the tracking of global norovirus transmission.

Appendix 1 Additional information about acute gastroenteritis associated with norovirus GII.8[P8], Thailand, 2023.

Appendix 2 Amino acid substitutions for investigation of acute gastroenteritis associated with norovirus GII.8[P8], Thailand, 2023.
