# Supplementary material for: Acute Gastroenteritis Associated with Norovirus GII.8[P8], Thailand, 2023
Source: Emerg Infect Dis. 2024 Jan;30(1):194–7. doi: 10.3201/eid3001.231264 (PMC10756372; doi:10.3201/eid3001.231264)
Supplement: Appendix 1 — Additional information about acute gastroenteritis associated with norovirus GII.8[P8], Thailand, 2023. [file 23-1264-Techapp-s1.pdf]

Article DOI: https://doi.org/10.3201/eid3001.231264

EID cannot ensure accessibility for supplementary materials supplied by authors. Readers who have difficulty accessing supplementary content should contact the authors for assistance.

Acute Gastroenteritis Associated with Norovirus GII.8[P8], Thailand, 2023

Appendix 1

Appendix 1 Table 1. Primers used to amplify the complete genomes of norovirus GII.8[P8]

ORF	Primer name	Sense	Sequence (5′-3′)	Position*
1	F1_GII.P8_1	F	GTGAATGAAGATGGCGTCTAAC	1–22
	1R_GII.P8_1180	R	CATGACCAATTTAAGTATGTCAAGG	1156–1180
	F2_GII.P8_968	F	TCGAACTTCATCGCGTCAC	968–986
	2R_GII.P8_2028	R	ATAGTCCCCTTCCCATGTGG	2009–2028
	F3_GII.P8_1913	F	CAACCAGACATGTGGAAGG	1913–1931
	3R_GII.P8_2924	R	CTGTGACGAGTCCCAGAAC	2906–2924
	F4_GII.P8_2752	F	CACAATAGAGGAGTACCTCCA	2752–2772
	4R_GII.P8_3912	R	TCCAATGAAGCACAGGCTT	3894–3912
	F5_GII.P8_3743	F	TCACTCCAACAAGTGATGC	3743–3761
	5R_GII.P8_4699	R	CTCAGTTTTGTCTGGCCTT	4681–4699
	RdRpF1_GII.P8_3380	F	ATGGGCATGCTGCTCACTG	3380–3398
	RdRp1R_GII.P8_4317	R	GTTGAGTCCCACCTAGAGTA	4298–4317
	RdRpF2_GII.P8_4049	F	ATGTACACAGCAGCCCTCAA	4049–4068
	RdRp2R_GII.P8_5165	R	CATGACCTCATGGTTGATCTC	5145–5165
2	F1VP1_GII.8_4967	F	ATCAAGAGTGGTGGTCTGGA	4967–4986
	1RVP1_GII.8_5659	R	CCAGCATTGTTAGCCCTTAGG	5639–5659
	F2VP1_GII.8_5403	F	ATAGTGCTTGCTGGGAATG	5403–5421
	2RVP1_GII.8_6393	R	CTGGCATGAACGAGCGGAA	6375–6393
	F3VP1_GII.8_6128	F	ACATGAGGCTAGGGTCAAC	6128–6146
	3RVP1_GII.8_6980	R	TGGTTGGAGCATTGATGGAAC	6960–6980
	F4VP1_GII.8_5786	F	ATCTGAGATGACAAATTCAAGAT	5786–5808
	F5VP1_GII.8_6127	F	CACATGAGGCTAGGGTCAA	6109–6127
3	VP2F1_GII.8_6616	F	ACTTCAGGTTTGAGGCATGG	6616–6635
	VP2R1_GII.8_7484	R	TCATTCTTTTCACTAAGCCCGTG	7462–7484
	VP2R2_GII.8_7497	R	ATAATCTAACCAAATCATTCTTTTCACTA	7469–7497

*Based on GII/Hu/JP/2002/GII.P8/SaitamaU25 (GenBank accession number AB039780).

Appendix 1 Table 2. Detailed clinical characteristics of individuals who tested positive for norovirus GII.8[P8]*

	Sample ID									
Parameter	B4899	B5182	B6213	B6941	B7634	B9202	B9256	B9804	B10039	B10069
Age (years)	5	7	6	12	29	3	12	10	12	7
Gender	Male	Male	Female	Male	Female	Male	Female	Male	Female	Female
Location	Saraburi	Bangkok	Nonthaburi	Nonthaburi	Bangkok	Bangkok	Chaiyaphum	Bangkok	Phuket	Phuket
Collection date	2018 Feb 02	2018 Feb 18	2018 Sep 18	2019 Jul 30	2020 Feb 04	2023 Feb 22	2023 Feb 27	2023 Apr 19	2023 Jun 14	2023 Jun 13
Setting	Hospitalized	Hospitalized	Hospitalized	Hospitalized	Hospitalized	Hospitalized	Outpatient	Hospitalized	Hospitalized	Hospitalized
Hospital stay (nights)	2	1	2	1	2	1	0	1	2	2
Diarrhea duration (days)	1	1	1	1	1	No	NI	1	3	No
Symptoms								Symptoms		
Diarrhea (per day)	5–6 times	NI	NI	2 times	1 time	No	No	NI	10 times	No
Vomiting (per day)	5–6 times	>10 times	3 times	No	No	3 times	5 times	5 times	5 times	>10 times
Nausea	No	No	No	No	Yes	No	Yes	Yes	NI	Yes
Abdominal pain	Yes	No	Yes	No	No	Yes	Yes	Yes	Yes	Yes
Fever	No	Yes	No	No	No	No	No	No	Yes	No
Headache	No	No	No	No	Yes	No	Yes	No	NI	NI
Vital signs								Vital signs		
Body Temperature (°C)	37	38.7	36.9	36.5	36.9	36.5	36.9	37.4	38	37.2
Pulse rate (per min.)	140	124	120	108	90	136	73	116	122	148
Respiratory rate (per min.)	20	20	22	20	20	22	18	20	24	24
Blood pressure (mmHg)	121/64	114/77	101/63	126/70	99/54	112/64	107/60	103/54	99/57	94/63
Serum leukocyte (cells/mm^3)	17,800	15,140	12,050	12,920	7,520	24,490	NI	15,350	10,160	13,310
Mode of transmission	NI	NI	NI	NI	NI	NI	NI	NI	Person-to-Person	Person-to-Person

*NI, no information.

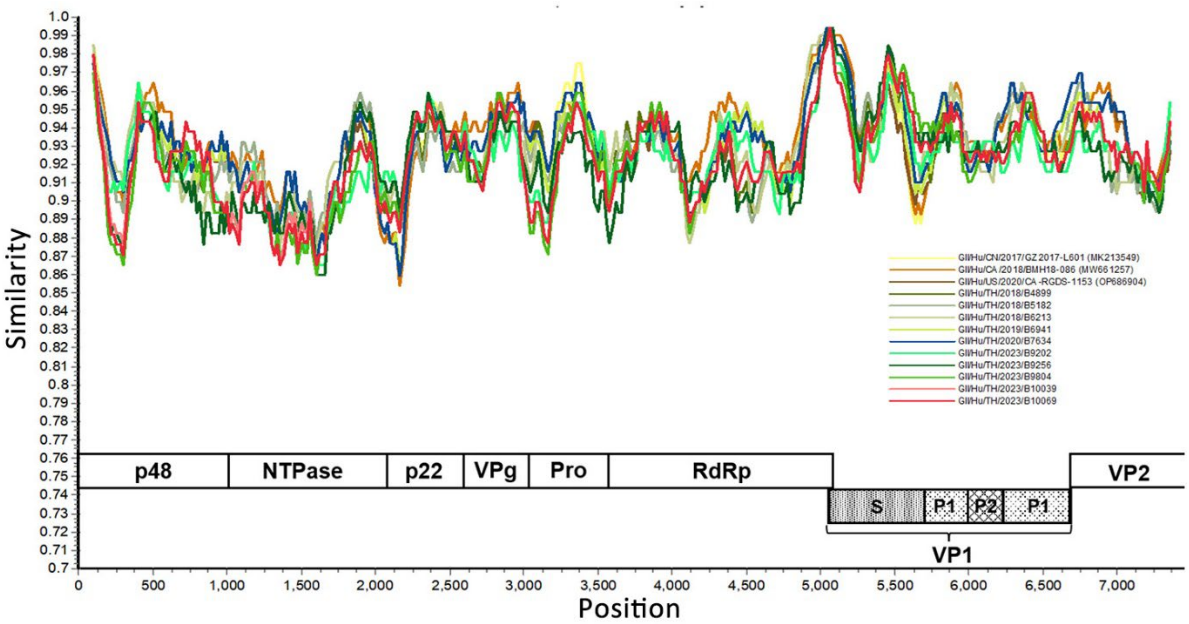

Appendix 1 Figure. Nucleotide sequence similarities among the ten GII.8[P8] Thai strains and three selected reference strains (GenBank accession numbers MK213549, MW661257, and OP686904) compared to the Norovirus Classification Working Group prototype GII/Hu/JP/2002/GII.P8/SaitamaU25 (AB039780) as query. Analysis was performed using SimPlot program version 3.5.1.
